# Effects of repetitive transcranial magnetic stimulation on children with low‐function autism

**DOI:** 10.1111/cns.13150

**Published:** 2019-06-22

**Authors:** Jian‐Nan Kang, Jia‐Jia Song, Manuel F. Casanova, Estate M. Sokhadze, Xiao‐Li Li

**Affiliations:** ^1^ College of Electronic & Information Engineering Hebei University Baoding China; ^2^ Department of Biomedical Sciences University of South Carolina School of Medicine Greenville Campus, Greenville Health System Greenville South Carolina; ^3^ State Key Laboratory of Cognitive Neuroscience and Learning and IDG/McGovern Institute for Brain Research Beijing Normal University Beijing China

**Keywords:** autism spectrum disorder, coherence, electroencephalography, neuromodulation, peak alpha frequency, rTMS

## Abstract

**Background:**

Autism spectrum disorder (ASD) is a very complex neurodevelopmental disorder, characterized by social difficulties and stereotypical or repetitive behavior. Some previous studies using low‐frequency repetitive transcranial magnetic stimulation (rTMS) have proven of benefit in ASD children.

**Methods:**

In this study, 32 children (26 males and six females) with low‐function autism were enrolled, 16 children (three females and 13 males; mean ± SD age: 7.8 ± 2.1 years) received rTMS treatment twice every week, while the remaining 16 children (three females and 13 males; mean ± SD age: 7.2 ± 1.6 years) served as waitlist group. This study investigated the effects of rTMS on brain activity and behavioral response in the autistic children.

**Results:**

Peak alpha frequency (PAF) is an electroencephalographic measure of cognitive preparedness and might be a neural marker of cognitive function for the autism. Coherence is one way to assess the brain functional connectivity of ASD children, which has proven abnormal in previous studies. The results showed significant increases in the PAF at the frontal region, the left temporal region, the right temporal region and the occipital region and a significant increase of alpha coherence between the central region and the right temporal region. Autism Behavior Checklist (ABC) scores were also compared before and after receiving rTMS with positive effects shown on behavior.

**Conclusion:**

These findings supported our hypothesis by demonstration of positive effects of combined rTMS neurotherapy in active treatment group as compared to the waitlist group, as the rTMS group showed significant improvements in behavioral and functional outcomes as compared to the waitlist group.

## 1 INTRODUCTION

Autism spectrum disorder ASD) is a very complex neurodevelopmental disorder characterized, according to the DSM‐5, by impairments of social interaction and communication skills, and repetitive behavioral patterns.^1^, ^2^ There are some other symptoms including the abnormal response to sensory stimuli, the difficulties in speech and language understanding and the lack of self‐care and social adaptation. The estimated prevalence was approximately 1 in 45 in United States according to the Centers for Disease Control and Prevention's National Center for Health Statistics report.^3^ The etiology of autism is very complex, and it is widely believed that the interaction between early environmental changes and genetic predisposition might be the cause of ASD.^4^

Several studies suggested that the impairment of GABAergic transmission might be the prime cause in the pathophysiology of autism,^5^ which acts as an inhibitory neurotransmitter in the mature brain for regulating neuronal excitability.^6^ In the early stages of brain development, abnormalities in GABAergic signaling might lead to aberrant information processing in ASD.^7^ Some studies have shown that some subtypes of autism are caused by the imbalance between the excitation and inhibition, which reflects primarily an inhibitory cortical deficit.^8^ Some studies showed that the excitatory/inhibitory bias was  caused by the abnormalities of minicolumns, which is the basic physiological and anatomical unit of the cerebral cortex.^9^, ^10^, ^11^ A directed stream of inhibition is imposed around the minicolumnar core by the double‐bouquet cells. The autistic children exhibit reduced size and increased number of cortical minicolumns, with a reduction and narrower width of the peripheral neuropil space. The physiological correlate of the described minicolumnopathy is a loss of surround inhibition and an alteration of the excitation and inhibition balance.^12^, ^13^ It is therefore unsurprising that autism is associated with inhibitory GABA neurotransmission abnormalities including reduced GABA_A_ and GABA_B_ subunit expression.^14^ The findings help explain the presence of seizures and sensory abnormalities in ASD.^15^ The imbalance might also relate to the manifestation of ASD symptoms including impaired cognitive ability, hyperactivity, poor language ability, and physical coordination.

A noninvasive neuromodulation technique, transcranial magnetic stimulation (TMS), is now widely used in the study of neurological diseases by altering the excitability of neurons and induces the cortex functional reorganization. It is based on the electromagnetic induction theory that a changing magnetic field can produce an electric current. Low‐frequency rTMS (≤1 Hz) can increase the inhibition of stimulated cortical regions, and high‐frequency rTMS (≥5 Hz) can increase the excitability of stimulated cortical regions.^16^, ^17^ rTMS is very safe if within safety guideline.^18^, ^19^ Many studies have also shown the effectiveness of rTMS intervention in autistic patients. Researchers believe this is due to a bias in the excitation and inhibition ratio of the cerebral cortex.^20^ Lower frequencies stimulation may preferentially induce currents along longitudinally oriented elements, that is, along axons rather than across the same.^21^ Accordingly, the disposition of interneurons and their projections so as to embrace the core of the minicolumns would make them especially susceptible to low‐frequency rTMS stimulation. A few studies supported that low‐frequency repetitive TMS can increase inhibitory activation of stimulated cortical regions.^22^ After receiving rTMS treatment to the dorsolateral prefrontal cortices DLPFC), autonomic balance was enhanced by facilitating frontal inhibition of limbic activity for the autistic children.^23^ Some ERP changes along with increased centro‐parietal P100 and P300 to targets are indicative of more efficient processing of information post‐TMS treatment,^24^ and significant reductions in both repetitive behavior and irritability were also found according to clinical behavioral questionnaires as a result of rTMS for ASD.^25^ Why choose DLPFC? The frontal lobe plays an important role in social, cognitive, and emotional functions. Some studies have shown that DLPFC is necessary for the operation of verbal/auditory information and non‐verbal/spatial information in working memory.^26^, ^27^, ^28^ Dysfunction in the anterior cingulate cortex and dorsolateral prefrontal cortex in ASD was identified by proton magnetic resonance spectroscopy.^29^ A large number of studies within the medical literature attest to a correlation between the activity of parvalbumin cells, gamma oscillations, and social deficits. Modulation of gamma oscillations, especially over the dorsolateral prefrontal cortex DLPFC), has been associated with improvements in cognitive performance.^30^, ^31^

Electroencephalography EEG) provides a resolution accurate to milliseconds to measure postsynaptic activity in the neocortex. Resting‐state EEG studies of ASD suggest a U‐shaped profile of electrophysiological power alterations, with excessive power in low‐frequency and high‐frequency bands.^32^ Multiscale entropy appears to go through a different developmental trajectory in infants at high risk for autism HRA) than it does in typically developing controls.^33^ Previous studies have shown that abnormalities in brain network connectivity were common in autistic children, which might lead to atypical interactions between brain regions that could lead to the social and cognitive impairment.^34^ For ASD, the ongoing changes of the pruning and synaptogenesis disturbed the normal brain development, thus leading to the abnormal neural connectivity.^35^ Therefore, differences in EEG signals can be used to compare the children with autism with the typical developmental children.^36^ Peak alpha frequency PAF) is an electroencephalographic measure of cognitive preparedness^37^ and might be a neural marker of cognitive function for the autism.^38^

The purpose of this study is to explore whether rTMS has positive effects on the brain activity and behavior of the autistic children. We proposed that low‐frequency rTMS over DLPFC will result in more pronounced improvements of functional outcomes as compared with waitlist group of ASD children, as we hypothesized that rTMS over the dorsolateral prefrontal cortex DLPFC) could improve excitation and inhibition ratio. In this study, the EEG data were recorded for every subject before and after 18‐sessions rTMS treatment. Autism Behavior Checklist ABC)^39^ was used to evaluate changes in behaviors including sensory, social skills, use of body and object, communication and language.

## 2 MATERIALS AND METHODS

### 2.1 Subjects

We enrolled 32 children (26 males and six females) with ASD, 16 children three females and 13 males; mean age: 7.8 years) received rTMS treatment twice every week, while the remaining 16 children matched with age and gender 3 females and 13 males; mean age: 7.2 years) were assigned to the waitlist group. All the children were all diagnosed with ASD by experienced psychiatrists in China based on the psychoeducational profile Third Edition) 40 and Diagnostic and Statistical Manual of Mental Disorders‐V criteria.^2^ A history of epilepsy, implants in the brain, and physical disability served as exclusionary criteria. We provided the informed consent to the parents of all participants and informed them the whole process before participation. The trial was conducted according to the Declaration of Helsinki and approved by the Beijing Normal University ethics committee.

### 2.2 rTMS treatment procedures

rTMS equipment used in this study was a Magstim *R^2^* stimulator (Magstim Company Limited). The motor threshold (MT) was identified before rTMS treatment for every subject. The center of a 70 mm figure‐eight coil was placed on the motor cortex of the subject, and the motor evoked potential (MEP) was recorded on the muscle of contralateral hand. When the induced amplitude was more than 50 μV in at least 5 out of 10 stimuli, the MT was determined.

The participants received 18 times rTMS treatment with two times per week.^23^, ^25^ During the first six times, the coil was placed over the left dorsolateral prefrontal cortex DLPFC), then the next six times it was placed over the right DLPFC, and the remaining six times it was placed on the bilateral DLPFC stimulation. Low‐frequency rTMS of 1 Hz and 90% MT was applied. There were 180 pulses each time with 18 trains with 10 pulses and an interval of 20 seconds.^41^ The waitlist group received the same process but the figure‐eight coil was placed vertically on the scalp with no magnetic field penetrated through the skull.

### 2.3 Behavioral evaluation

In this study, the scores of Autism Behavior Checklist (ABC) were recorded before and after the rTMS treatment. The ABC was filled out by their parents and serves to screen for a number of behaviors. There are 57 sub‐items in the ABC, each one being scored; generally, the higher scores represent more serious behavioral problems.

### 2.4 EEG data collection and analysis

For participants, EEG data were recorded two times, one was before rTMS treatment and the other was after 18 sessions of rTMS treatment. For the waitlist group, EEG data were also recorded two times. EEG data were collected 20 min before rTMS and 20 min after rTMS in a quiet room, and the participants were awake and relaxed with eyes‐open state. We used 5 cm rule to find the DLPFC in each patient. We tried to use the navigation system to determine the location, but the autistic children did not cooperate and we could not complete. During the data‐recording process, 5 minutes resting‐state EEG data were recorded with a 128 HydroCel Sensor Net System (Electrical Geodesics, Inc), and the central vertex was set as the reference electrode. The impedances were controlled less than 50KΩ for all the channels, and the sampling rate was 1000 Hz. Sixty‐two electrodes were selected for faster and more accurate results, 14 EEG sites for frontal lobe, 15 EEG sites for central lobe, 8 EEG sites for left temporal lobe, 8 EEG sites for right temporal lobe, and 17 EEG sites for occipital lobe.

**Figure 1:**
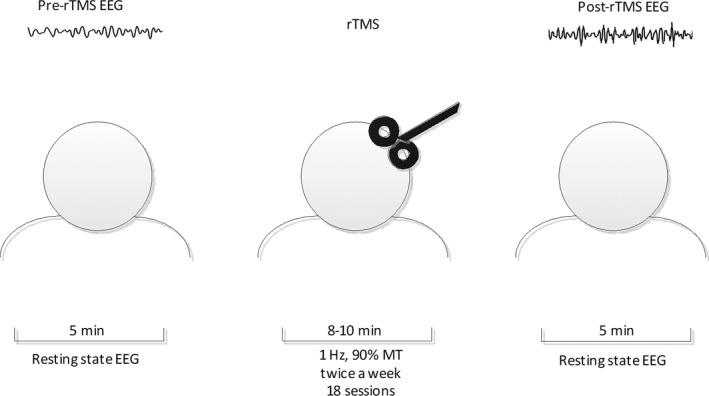
Schematic presentation of rTMS modified from.^41^ About 5 min, EEG data were recorded before and after 18 sessions. Every participant received 18 sessions twice a week (1 Hz, 180 pulses every time)

Matlab R2016a and EEGlab V13.5.4b were used for off‐line data analysis. Data preprocessing was also done by removing 50 Hz power frequency and filtering between 0.5 and 45 Hz. Artifacts eye blink, eye movement, and muscular artifact) were rejected from analyses by computer selection and visual inspection. Data were segmented for continuous segments of 4 seconds 4000 data points: 4 seconds × 1000 Hz). Figure 1.

### 2.5 Peak alpha frequency

The data were divided into eight sections of the same length with 50% overlap and scanned with a Hamming window. Spectral density was calculated by fast Fourier transform FFT). In this study, relative power was used to perform the analysis. EEG was divided into five frequency band including delta 1‐4 Hz), theta 4‐8 Hz), alpha 8‐13 Hz), beta 13‐30 Hz), and gamma 30‐45 Hz). Relative power was the amount of EEG activity in one frequency band. The primary EEG measurement was the individual peak alpha frequency iPAF) for each of 62 electrode sites, which was defined as the alpha frequency where the maximum power occurs.^42^

### 2.6 Coherence

Coherence provides a measure of the degree of synchronization between two signals, which means that the two signals with the same frequency have the consistent phase relationship over time, and we could also assume there is a high degree of the coordinated brain activity between the underlying brain areas where those two signals come from.^35^ EEG coherence is one way to assess the brain functional connectivity, which has proven abnormal in previous studies for ASD children.^43^, ^44^ In this study, we calculated the coherence of 62 channels at the alpha frequency band. First, one channel was selected and the correlation coefficient to other channels was obtained. Then, we obtained coherence estimation of the square of the input signal amplitude. The formula is $C_{\mathrm{xy}}=\frac{{\left|P_{\mathrm{xy}}\right|}^{2}}{P_{\mathrm{xx}}P_{\mathrm{yy}}}$. Its value was between 0 and 1, which showed the correlation degree of each channel.

## 3 RESULTS

In the current study, we tried to find rTMS effects on the brain activity and behavior for ASD children. First, we calculated the iPAF for each of 62 electrode sites before and after rTMS treatment and paired t‐test statistical analysis and the correction for multiple contrasts were used.

**Figure 2:**
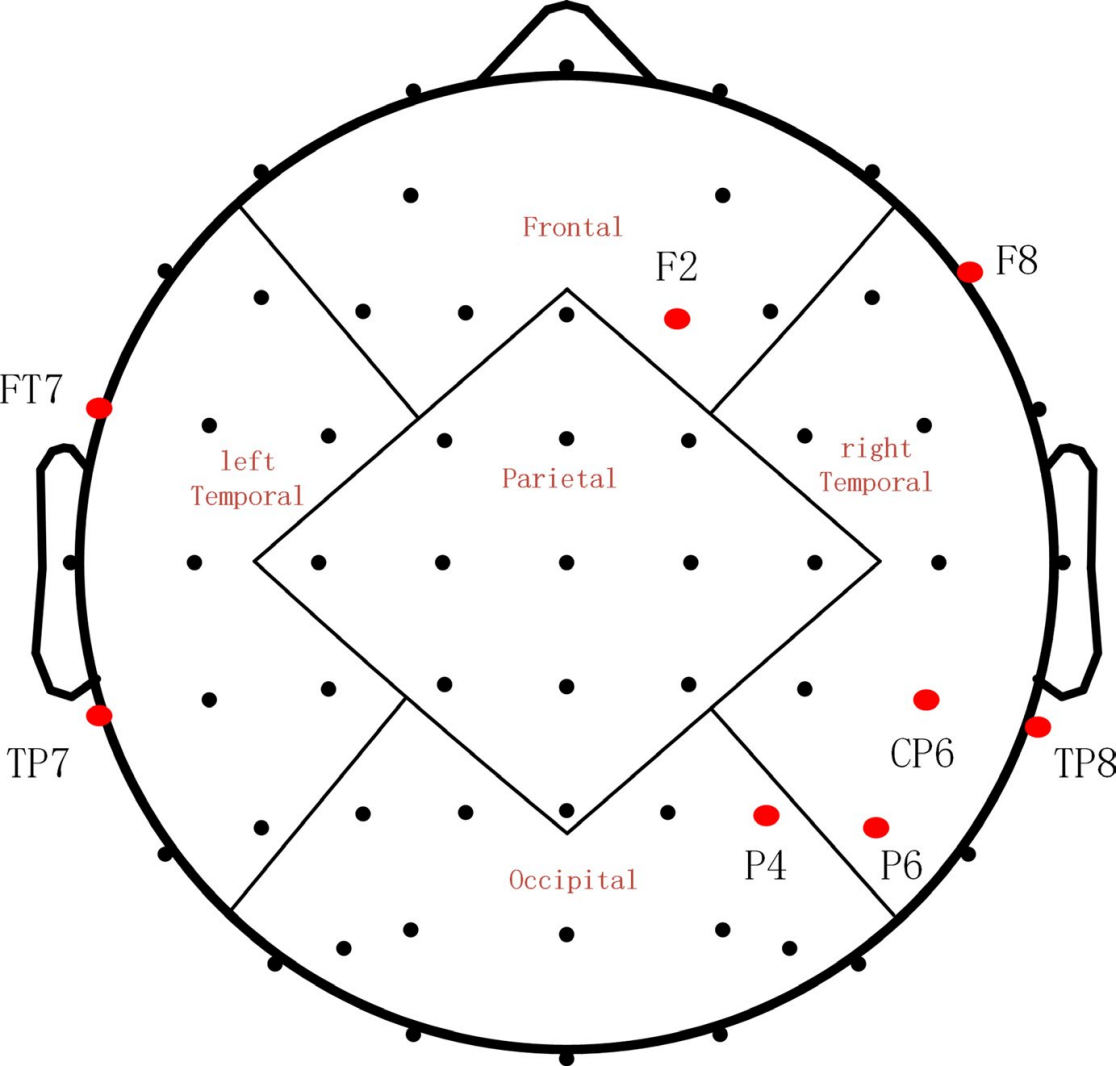
Changes of iPAF after rTMS treatment. Significant increases were found in iPAF after rTMS treatment shown by red circles (*p < 0.05)

There are significant increases in the iPAF from four brain regions after receiving rTMS treatment including Frontal (F2 (*t* = −2.195, *p* = 0.046), F8 (*t* = −2.527, *p*= 0.024)), right Temporal (CP6 (*t* = −2.552, *p*= 0.023), TP8 (*t* = −2.506, *p* = 0.025)), Occipital (P6 (*t* = −3.022, *p* = 0.009), P4 (*t* = −2.492, *p*= 0.026)), and left Temporal (FT7 (*t* = −2.261, *p* = 0.040), TP7 (*t* = −2.337, *p* = 0.035)) Table 1 and Figure 2, and we found no significant differences for waitlist group who have not received rTMS treatment.

**Table 1:** The significant iPAF change before and after rTMS

	Before	After	
F2	9.4186	9.4858	↑
F8	9.3629	9.4711	↑
FT7	9.3613	9.4723	↑
TP7	9.4032	9.5110	↑
CP6	9.3823	9.4991	↑
TP8	9.3950	9.4956	↑
P4	9.4042	9.4638	↑
P6	9.4136	9.5216	↑

In order to measure the changes of brain functional connectivity before and after rTMS for the participants, we calculated the coherence for every two of 62 channels at alpha frequency band. The results showed a significant increase of coherence between Cz‐T8 (*P* = 0.038) and CPz‐C4 (*P* = 0.023) after rTMS treatmentFigure 3, and no significant differences were found for waitlist group who have not received rTMS treatment. We also calculated the coherence in other bands but found no significant differences.

**Figure 3:**
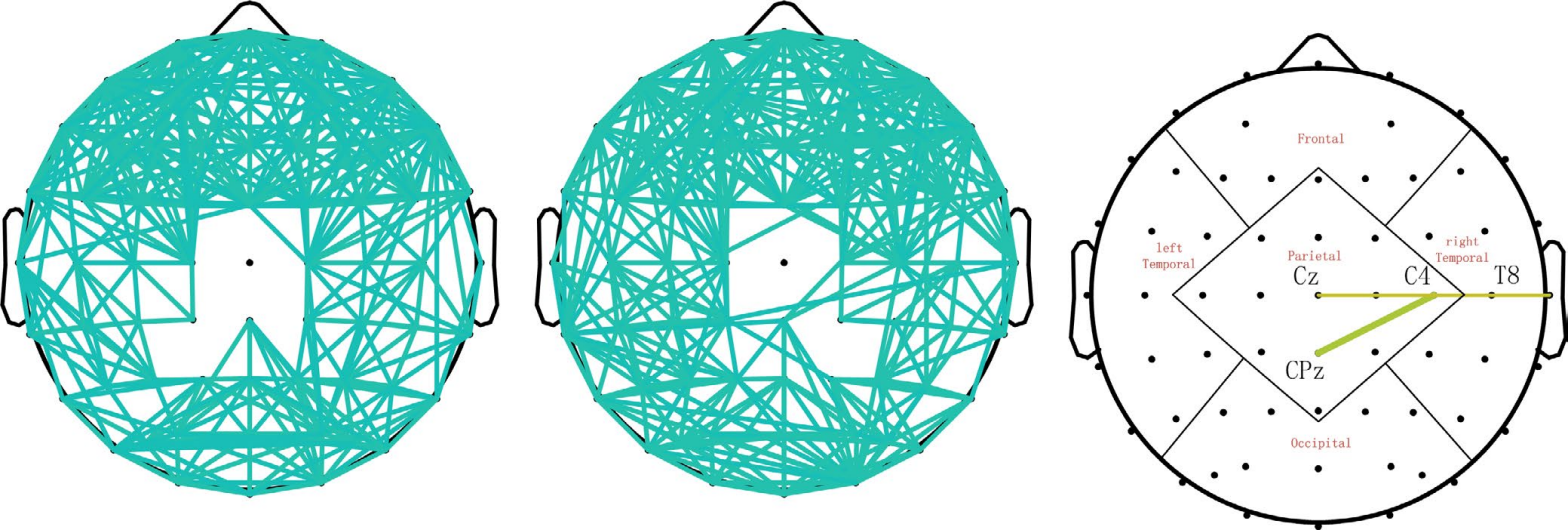
Changes of the coherence at alpha band for participants. *p < 0.05. (A) showed the coherence before receiving rTMS treatment; (B) showed the coherence after receiving rTMS treatment; (C) showed significant increase of coherence between Cz‐T8 and CPz‐C4 after rTMS treatment

**Figure 4:**
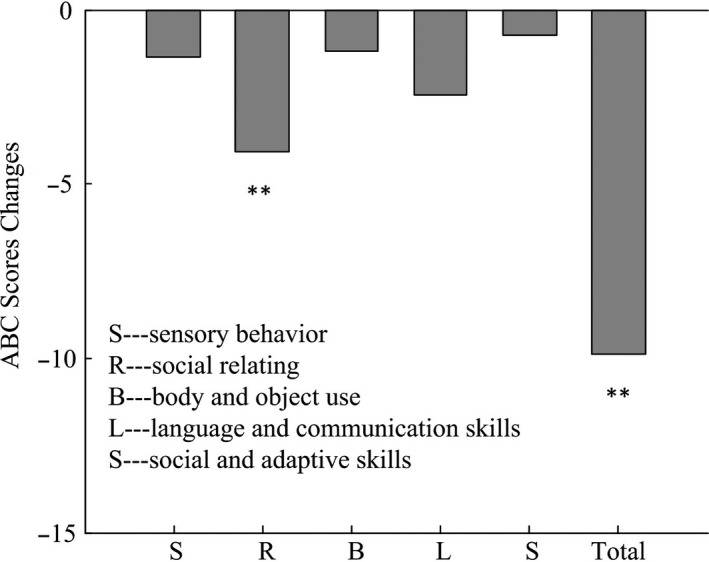
Changes of Autism Behavior Checklist (ABC) scores after rTMS as compared to the baseline levels in children with ASD, **p< 0.01

Finally, we also compared ABC scores changes before and after rTMS to evaluate the behavioral changes of the participants. The details of ABC scores are shown in Table 2. There were significant changes of ABC scores including social relating behaviors and total scores for the participants Figure 4.

**Table 2:** The scale score of each subject before and after rTMS(**p<0.01)

	*S*		R**		*B*		*L*		*S*		Total**	
S1	24/20	↓	25/23	↓	27/31	↑	19/8	↓	10/16	↑	105/98	↓
S2	4/6	↑	13/5	↓	0/3	↑	6/11	↑	18/15	↓	41/40	↓
S3	14/15	↑	13/11	↓	18/5	↓	26/22	↓	18/13	↓	89/66	↓
S4	7/6	↓	18/11	↓	9/3	↓	10/6	↓	9/7	↓	53/33	↓
S5	22/20	↓	22/17	↓	2/5	↑	13/18	↑	9/6	↓	68/66	↓
S6	15/8	↓	15/7	↓	6/4	↓	5/10	↑	11/13	↑	52/42	↓
S7	12/14	↑	11/10	↓	3/4	↑	12/9	↓	8/10	↑	47/47	‐‐
S8	0/0	‐‐	0/0	‐‐	14/11	↓	6/8	↑	12/5	↓	32/24	↓
S9	14/10	↓	16/9	↓	0/0	‐‐	5/4	↓	5/7	↑	40/30	↓
S10	17/16	↓	15/12	↓	0/2	↑	10/9	↓	16/15	↓	58/54	↓
S11	12/10	↓	11/11	‐‐	26/22	↓	9/10	↑	12/10	↓	70/63	↓
S12	18/20	↑	24/13	↓	4/4	–	14/10	↓	12/16	↑	72/63	↓
S13	8/10	↑	13/9	↓	0/2	↑	12/4	↓	8/8	–	41/33	↓
S14	18/11	↓	19/20	↑	28/26	↓	16/6	↓	19/15	↓	100/78	↓
S15	21/21	↑	14/12	↓	22/16	↓	12/8	↓	11/12	↑	80/69	↓
S16	7/4	↓	13/7	↓	0/2	↑	13/6	↓	12/10	↓	45/29	↓

## 4 DISCUSSION

The outcome of behavioral evaluations using ABC questionnaire showed improvements in autism symptoms similar to previous study results.^24^ Our results also showed there were significant increases in the iPAF from eight electrode sites after receiving rTMS treatment. The distributed brain areas include the left temporal region, the right temporal region, and the occipital region. There was evidence that PAF was associated with cognitive performance at the general level of intelligence which was found to associate with a broad range of cognitive tasks^45^ and with nonverbal cognitive functioning in ASD.^38^ PAF is also an important indicator of brain maturation and brain network development during childhood.^46^, ^47^ For ASD children, the normal increase in PAF with age was not found 48 but rather abnormal signs of neuromaturation, brain structure, and functional development were found.^49^

We also found a significant increase of alpha coherence between the central lobe and the right temporal lobe which showed the improvements of cognitive arousal level and brain functional.^50^ Coherence provides a measure of the degree of synchronization between two signals, and we could assume there is a high degree of the coordinated brain activity between the underlying brain areas. It is an effective algorithm to measure the functional connection of the brain. Some previous research showed that the atypical functional connectivity has been found as a core feature in ASD.^51^, ^52^ The repetitive behaviors were found to correlated with functional connectivity in the posterior cingulate cortex, medial frontal cortex, medial temporal lobes, and the superior frontal gyrus in ASD patients, the lower the degree of functional connection, the more severe the impairments.^53^ Overall, the results showed positive effects on EEG activity after receiving rTMS treatment for the autistic children.

It was feasible to select DLPFC as a site for rTMS stimulation because the dysfunction in the anterior cingulate cortex and dorsolateral prefrontal cortex and disruption in the ratio between cortical excitation and inhibition especially within the prefrontal cortex in individuals with autism were identified.^54^ An imbalance of excitation and inhibition ratio could adversely affect patterns of cortical activation. A course of 18 neuromodulatory sessions of low‐frequency rTMS might restore the cortical excitation and inhibition ratio balance by selective activation of double‐bouquet cells at the periphery of cortical minicolumns, and it was shown that minicolumnar abnormalities in autism are most significant within the prefrontal cortex, more specifically, the DLPFC.^8^

In a recent review on use of TMS in ASD,^55^ TMS could be particularly informative in detecting abnormalities in excitation and inhibition ratio in ASD given theoretical studies regarding role of GABAergic interneurons in autism etiology and specifically role of high excitation and inhibition ratio balance in autism. Previous studies have also shown that rTMS effects are mediated by fronto‐limbic connections, which is a complex structural network that is the center of anxiety and emotion regulation.^56^ Low‐frequency rTMS over the DLPFC could increase the activation of inhibitory circuits and lower the ratio of cortical excitation to inhibition leading to a better control of limbic function 57, 58 and corresponding behavioral changes.

But there are some limitations in this study. First, we used a waitlist group as a control group rather than using a randomized clinical trial design with a sham rTMS. We considered this study as a preliminary pilot with a WTL group design and plan to consider progression to a randomized clinical trial design in the future. Second, the sample size is relatively small. We are going to continue our experiment and expand the sample size in the next stage.

In conclusion, the study showed that treatment with prefrontal low‐frequency rTMS improved brain functioning and behavioral symptoms in autism. rTMS might be a better alternative for patients with ASD who are not suitable for the psychopharmacological treatments. This study provides support to the statement that rTMS can be regarded as perspective neuromodulatory treatments targeting symptoms of ASD. However, further research on the dose, type, and exact parameter setting is needed to investigate the potential of rTMS treatment and to explore underlying mechanisms.
